# Upper Gastrointestinal (GI) Manifestations of Inflammatory Myositis: A Tale of Two Patients

**DOI:** 10.7759/cureus.62153

**Published:** 2024-06-11

**Authors:** Knkush Hakobyan, Talar Acob, Mesrop Aleksanyan, Omar Jumaah, Sajina Prabhakaran

**Affiliations:** 1 Internal Medicine Residency Program, Capital Health Regional Medical Center, Trenton, USA; 2 Oncology, Yerevan State Medical University, Yerevan, ARM; 3 Rheumtology, Capital Health Regional Medical Center, Trenton, USA

**Keywords:** intravenous immunoglobulins (ivig), oropharyngeal dysphagia, s: dermatomyositis, polymyositis myalgia, academic rheumatology

## Abstract

Myositis is a group of rare autoimmune disorders characterized by chronic inflammation of skeletal muscles that leads to a hallmark triad of muscle weakness, fatigue, and myalgia. Extra-muscular manifestations are sometimes seen and involve various organ systems, including the gastrointestinal (GI) tract. In this case series, two patients with polymyositis (PM) and dermatomyositis (DM), both of whom developed dysphagia as a complication of myositis, are discussed. Case 1 was a female with a known history of biopsy-proven dermatomyositis who presented with progressive peripheral edema and weakness affecting all extremities. Concurrently, she displayed symptoms of pneumonia and dysphagia associated with frequent spontaneous or self-induced vomiting to alleviate retrosternal discomfort. Esophagogastroduodenoscopy (EGD) revealed esophageal dilatation and an absence of a contractile response, consistent with myositis. Treatment comprised intravenous immunoglobulin (IVIG), mycophenolate, and lifestyle modifications, including dietary adjustments and maintaining an upright position postprandial. The second case was a female with muscle weakness and dysphagia. Video-fluoroscopic swallow assessment was significant for pharyngeal dysfunction without a sensory response to penetrated material, and the patient was at high risk of aspiration with any oral intake. The presence of pharyngeal dysfunction and dysphagia prompted treatment with IVIG, mycophenolate, and percutaneous endoscopic gastrostomy (PEG) tube placement. These cases have highlighted the upper GI complications observed in patients with myositis, accentuating the necessity for a personalized treatment approach. Timely intervention has shown promising results in symptomatic relief and improving patient outcomes. This emphasizes the importance of a multidisciplinary approach when addressing myositis-related upper GI manifestations.

## Introduction

Inflammatory myopathies, also known as myositis, are a group of rare autoimmune disorders characterized by chronic inflammation of skeletal muscles that leads to a hallmark triad of muscle weakness, fatigue, and myalgia. Five main types of inflammatory myopathies are now widely differentiated: dermatomyositis (DM), immune-mediated necrotizing myopathy, sporadic inclusion-body myositis, overlap myositis (including anti-synthetase syndrome), and polymyositis (PM) [1]. Extra-muscular manifestations are sometimes seen and involve various organ systems, including the gastrointestinal (GI) tract. Dysphagia, secondary to esophageal smooth muscle involvement, is a common GI manifestation of myositis and is observed in up to 40% of patients with myositis [2]. In this case series, two patients with PM and DM, both of whom developed dysphagia as a complication of myositis, are discussed.

## Case presentation

Case 1

**Figure 1:**
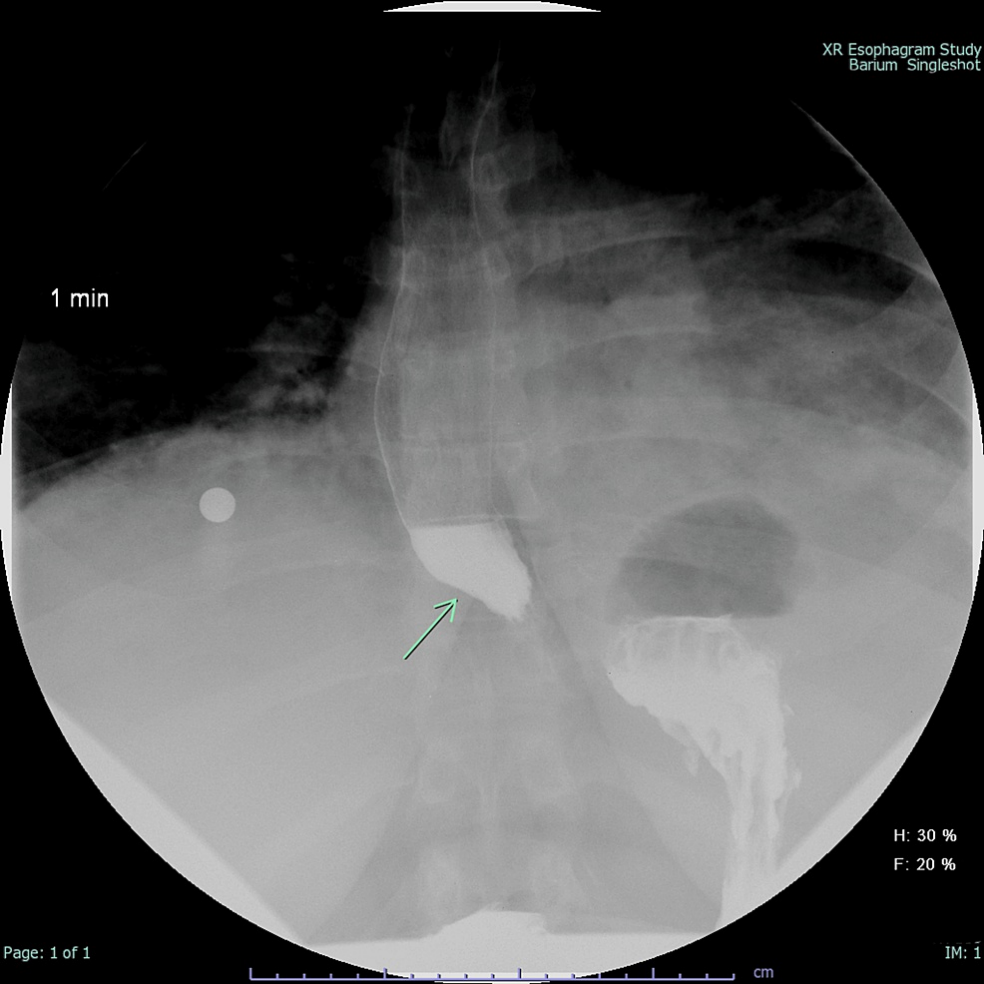
XR esophagogram of patient 1 The green arrow shows dilatation of the distal esophagus and mild narrowing of the gastroesophageal junction. Characteristics of achalasia.

A 32-year-old female with a known history of biopsy-proven dermatomyositis, whose biopsy showed severe active myopathy of the left leg and a perifascicular pattern of myocyte injury in major histocompatibility complex (MHC) class I and II upregulation morphologically consistent with dermatomyositis, presented with progressive peripheral edema and weakness affecting all extremities. Additionally, she displayed symptoms of dysphagia associated with frequent spontaneous or self-induced vomiting to alleviate retrosternal discomfort. Primary workup revealed elevated serum creatinine phosphokinase, aspartate aminotransferase (AST), alanine transaminase (ALT), and inflammatory markers (erythrocyte sedimentation rate (ESR), C-reactive protein (CRP)). Serological testing revealed a high anti-Jo1 antibody titer level. During her initial hospital stay, the patient was started on IV Solu-Medrol 20 mg two times daily. After discharge, the patient lost follow-up and was not compliant with treatment for dermatomyositis, which included prednisone and mycophenolate mofetil (Figure 1).

**Figure 2:**
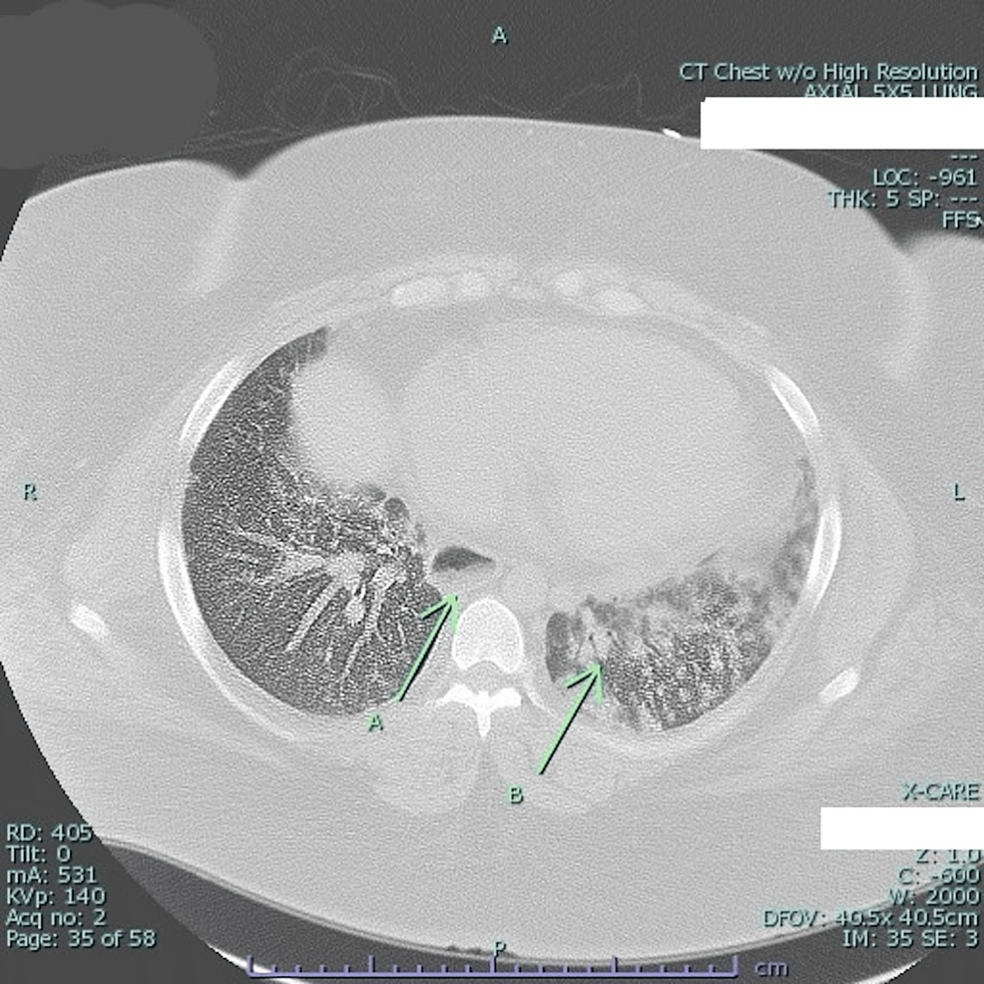
CT of chest without contrast Green arrow A: small amount of debris in distal esophagus; Green arrow B: airspace consolidation with groundglass opacities.

In a short period of time, the patient was hospitalized again due to shortness of breath and was diagnosed with pneumonia. She improved with broad-spectrum antibiotics. Chest CT revealed stable interstitial lung disease secondary to DM, and an incidental finding of a patulous esophagus displaying debris was noted (Figure 2), correlating with her history of dysphagia and regurgitation. XR esophagogram swallow assessment suggested mild achalasia and duodenal diverticula. A 2D ecocardiogram showed elevated pulmonary artery pressure (51 mmHg). Subsequently, esophagogastroduodenoscopy (EGD) revealed esophageal dilatation and an absence of a contractile response consistent with myositis. Pulmonary function tests (PFTs) are indicative of severe interstitial lung disease (ILD). 

During this period, the patient was encouraged to adopt dietary modifications, such as consuming small, frequent meals and avoiding lying down immediately after eating. Her medication regimen was continued with mycophenolate mofetil at 3000 mg/day, prednisone at 8 mg/day, and weekly alendronate, supplemented by vitamin D. Cardiac evaluation with a 2D echocardiogram showed resolution of previously elevated right heart pressures. Despite treatment adherence, her myositis symptoms persisted, prompting the addition of intravenous immunoglobulin (IVIG) at 2 g/kg divided over three days, to be repeated every four weeks. Preventative measures, including vaccinations for influenza virus, COVID booster, shingles, and pneumonia, were also recommended.

On follow-up, the patient reported significant improvement in myositis symptoms, including muscle weakness with IVIG infusions, resolution of dysphagia, and no longer experiencing heartburn. However, she continued to experience exertional limitations. 

Case 2

**Figure 3:**
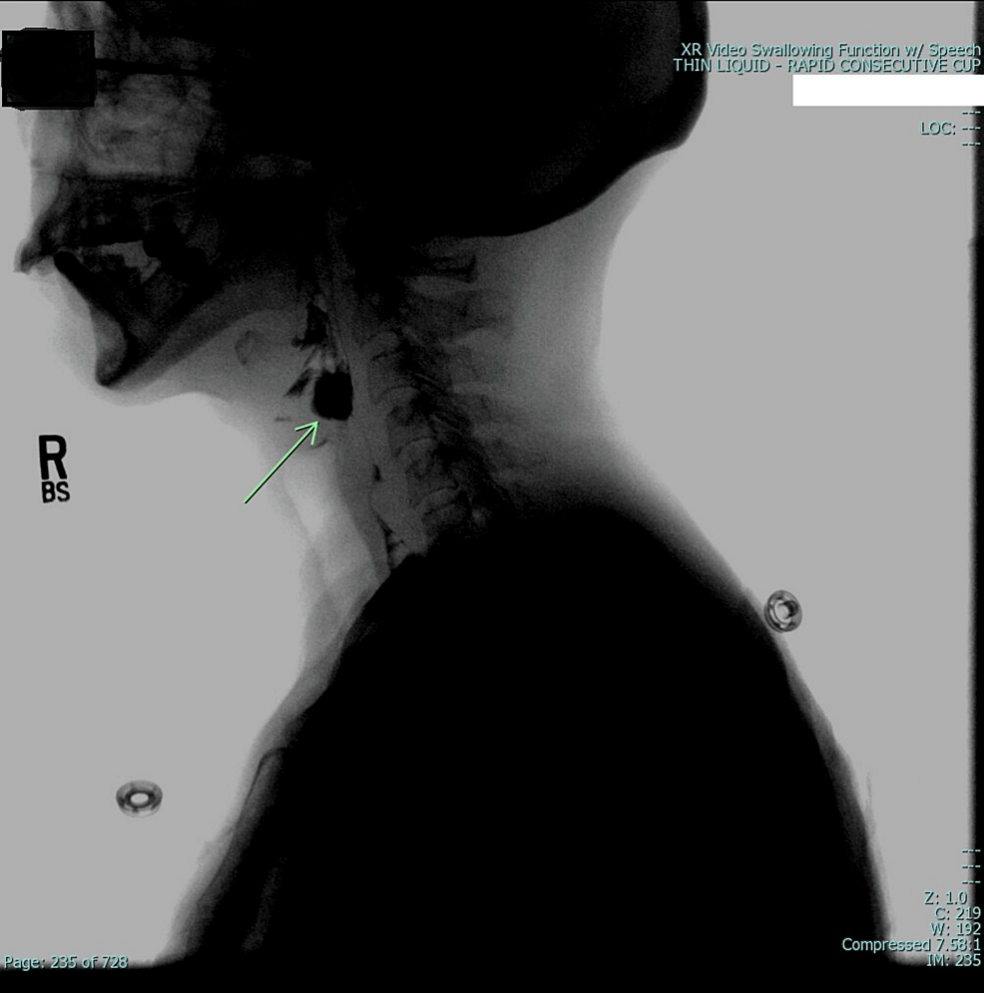
Video-fluoroscopic study Green arrow: shows penetration without aspiration drinking thin liquid.

The second patient is a 59-year-old female with a known history of skin biopsy-proven scleroderma who was admitted to our facility with complaints of severe muscle weakness and dysphagia. Lab work showed positive anti-polymyositis antibodies and weak positive anti-Mi2 antibodies, raising the suspicion of a multifactorial etiology. A deltoid muscle biopsy showed inflammatory myopathy and a perifascicular pattern of myocyte injury, confirming the diagnosis of polymyositis in addition to scleroderma. The patient underwent a video-fluoroscopic swallow assessment (Figure 3), which revealed significant pharyngeal dysfunction without a sensory response to penetrated material, placing her at a high risk of aspiration with any oral intake. Esophagogastroduodenoscopy (EGD) demonstrated absent proximal peristalsis, although no evidence of esophageal strictures characteristic of scleroderma esophagus was found.

Given the presence of pharyngeal dysfunction and dysphagia, the patient was initiated on treatment with intravenous immunoglobulin (IVIG) 1 g/kg divided over three days and mycophenolate 1000 twice daily. A percutaneous endoscopic gastrostomy (PEG) tube was inserted for enteral feeding, which remained in place following discharge. During the follow-up visit, the patient, previously reliant on a wheelchair, exhibited improved muscle strength and was able to stand up unassisted and ambulate a few steps.

In light of the patient's progressive recovery, a transition to liquid feeding alongside tube feedings was initiated. In further follow-up appointments, the PEG tube was removed, and the patient was able to achieve a regular diet.

## Discussion

Dysphagia, secondary to esophageal smooth muscle involvement, is a common GI manifestation of myositis and is observed in up to 40% of patients. Dysphagia in patients with myositis is usually a symptom of reduced endurance of swallowing musculature rather than mechanical obstruction of the upper esophageal sphincter [3]. Treatment modalities, including IVIG, have promising effects on improving dysphagia symptoms in patients with different types of myositis. In this case series, two patients with advanced myositis are presented.

The first case presents a multiorgan involvement clinical picture involving dermatomyositis, interstitial lung disease (ILD), and gastrointestinal dysmotility secondary to her autoimmune condition. The complexity of her condition necessitated a comprehensive and multidisciplinary approach to management.

One significant challenge in her management was medication adherence. Her non-adherence during a critical period led to a worsening of symptoms, emphasizing the need for continuous patient education and support. Additionally, her treatment regimen, involving mycophenolate mofetil, methotrexate, and prednisone, required careful monitoring for potential adverse effects and drug interactions.

The positive response to IVIG indicated an effective management strategy for her muscle inflammation. IVIG is a safe and effective treatment against both muscular and extra-muscular manifestations of myositis [4]. Additionally, dietary modifications and weight management were crucial for her overall health and to reduce systemic inflammation.

The second case highlights the complex interplay between scleroderma and polymyositis (PM) in a patient presenting with severe muscle weakness and dysphagia. Scleroderma, a chronic systemic autoimmune disease characterized by fibrosis of the skin and internal organs, can lead to significant clinical symptoms. Polymyositis, another autoimmune condition characterized by inflammation and degeneration of muscle tissue, coexists with scleroderma, complicating the clinical picture.

The treatment regimen of IVIG and mycophenolate was chosen due to their immunomodulatory effects, aiming to reduce muscle inflammation and improve strength. The PEG tube placement was a necessary intervention to ensure adequate nutrition and prevent aspiration, given the high risk identified during the swallowing assessment.

The patient's significant improvement in muscle strength and ambulation at follow-up underscores the importance of early and aggressive treatment in managing autoimmune overlap syndromes. The gradual introduction of liquid feeding alongside PEG tube feedings represents a cautious approach to reintroducing oral intake, prioritizing patient safety and nutritional status [5].

## Conclusions

These cases have highlighted the upper GI complications such as achalasia and dysphagia observed in patients with myositis, accentuating the necessity for a personalized treatment approach. Timely intervention has shown promising results in symptomatic relief and improving patient outcomes. This emphasizes the importance of a multidisciplinary approach when addressing myositis-related upper GI manifestations.
